# Clinical and radiographic outcomes of long monoblock, hydroxyapatite-coated stem in revision hip arthroplasty with extended trochanteric osteotomy: a multicenter study

**DOI:** 10.1186/s13018-023-04377-1

**Published:** 2024-01-03

**Authors:** Peng Xin, Jianfeng Yang, Guangxing Chen, Yiming Wang, Yan Wang, Guoqiang Zhang

**Affiliations:** 1 Department of Orthopedics, The First Medical Center of Chinese, PLA General Hospital, Beijing, China; 2 Senior Department of Orthopedics, The Fourth Medical Center of Chinese PLA General Hospital, Beijing, China; 3 Department of Orthopedics, Chinese PLA Southern Theater Command General Hospital, Guangzhou, China; 4 Department of Orthopedics, The Southwest Hospital of Army Medical University, Chongqing, China; 5 Medical School of Chinese People’s Liberation Army, Beijing, China

**Keywords:** Revision total hip arthroplasty, Corail revision stem, Extended trochanteric osteotomy, Clinical outcomes, Radiographic outcomes

## Abstract

**Background:**

The distally fixed stem used in revision total hip arthroplasty (rTHA) with extended trochanteric osteotomy (ETO) is subject to periprosthetic fracture, stem subsidence, and stress shielding. The prospective multicentric study aimed to assess the clinical and radiographic outcomes, and complications of using the Corail revision stem in rTHA with ETO.

**Methods:**

Sixty-four patients undergoing rTHA with ETO using the Corail revision stem between 2019 and 2020 were enrolled in the study. We performed a postoperative follow-up of the patient and obtained radiographs and Harris hip scores (HHSs). These results were used to analyze ETO union, Engh scores, bone remodeling, stem stability and hip function.

**Results:**

The mean follow-up duration was 34 months (range 23–41). Sixty-two patients who underwent ETOs achieved complete healing at the final follow-up. Fifty-nine hips had bony ingrowth from the osteotomy fragment to the stem without radiolucent lines. The postoperative Engh score was 21.3 ± 3.59 (range 15.5–27.0). Forty-three hips had regeneration in the proximal femur. Two patients had transient thigh pain postoperatively. The postoperative HHS improved from 40.7 ± 16.67 (range 0–67) preoperatively to 82.1 ± 6.83 (range 73–93).

**Conclusion:**

Corail revision stems are a viable and reliable option in rTHA with ETO. This stem had excellent clinical and radiographic outcomes, resulting in a high rate of ETO union and stem survival. The revision stem enabled restoration of proximal bone stock in femurs with prerevision bone defects, which were prepared for the next revision operation.

*Level of evidence* Level IIb, Prospective self-control study.

## Introduction

Extended trochanteric osteotomy (ETO) is often used in revision total hip arthroplasty (rTHA) to remove the prosthesis and protect the femoral cavity, which has the advantages of good exposure, correction of deformity and easy healing [1–4]. As ETO results in diminished proximal femoral support, distally fixed stems are often implanted for femoral reconstruction and to achieve immediate and reliable distal fixation [5–9]. However, distally fixed stems are prone to complications such as femoral stress shielding, periprosthetic fracture, prosthesis subsidence [10, 11], nonunion, and fracture or migration of the osteotomy fragment [5, 12–14].

These complications frequently occurred in revision THA. The incidence of femoral stress shielding is 22 to 50% [15–17]. Amanatullah et al. [16] and Feng et al. [18] reported that the incidence of intraoperative femoral fracture during insertion of the stem was 12% and 17%, respectively. Parry et al. [19] reported that significant subsidence occurred in 13% of stems with an average amplitude of 18 mm. Abdelsamie et al. [20] found that subsidence was significantly associated with ETO. Garabano et al. [5] found that ETO nonunion rate was 15%. Abdel et al. [12] reported that 7% of ETO fragment migrated larger than 1 cm. Since the distal fixed stem is secured in the medullary isthmus and the load transmission nonphysiologically bypasses the proximal femur, resulting in stress shielding. Stress shielding results in young patients who may not have sufficient bone stock for future revision surgery, making revision surgery more difficult [21]. To solve the above problems and obtain better outcomes, some surgeons have focused their attention on a long monoblock, fully hydroxyapatite (HA)-coated femoral stem (Corail revision stem, DePuy, Leeds, UK). Implantation of this stem involves “mixed distally and proximally fixed” and “fit and fill,” achieving good clinical results. Saunders et al. [22] found that this stem has good 6-year survival, acceptable complication rates, adequate proximal bone loading, bone ingrowth and remodeling, little subsidence, and reliable clinical performance in revision hip arthroplasty. Chatelet’s research reached a similar conclusion at a minimum follow-up of 5 years [23]. However, this stem used in rTHA with ETO has not been studied, even though ETO fragments can be stabilized with the existing fixation method to allow adequate support of the proximal femur [7].

The objective in this multicentric study was to investigate the clinical and radiographic outcomes, as well as survival, complications of the long monoblock, fully HA-coated femoral stem in rTHA with ETO. Our hypothesis was that this revision stem can be used in rTHA with ETO, and ETO union, regeneration, and osseointegration might be confirmed in the proximal femur.

## Materials and methods

### Patient cohort

This prospective study was a multicenter study, involving four surgeons at three study centers. The study received institutional review board approval, was registered at the Chinese Clinical Trial Registry, and adhered to Consolidated Standards of Reporting Trials guidelines. The protocol for the study was approved by the Ethics Committee. Written informed consent was obtained from all participants prior to surgery.

**Fig. 1:**
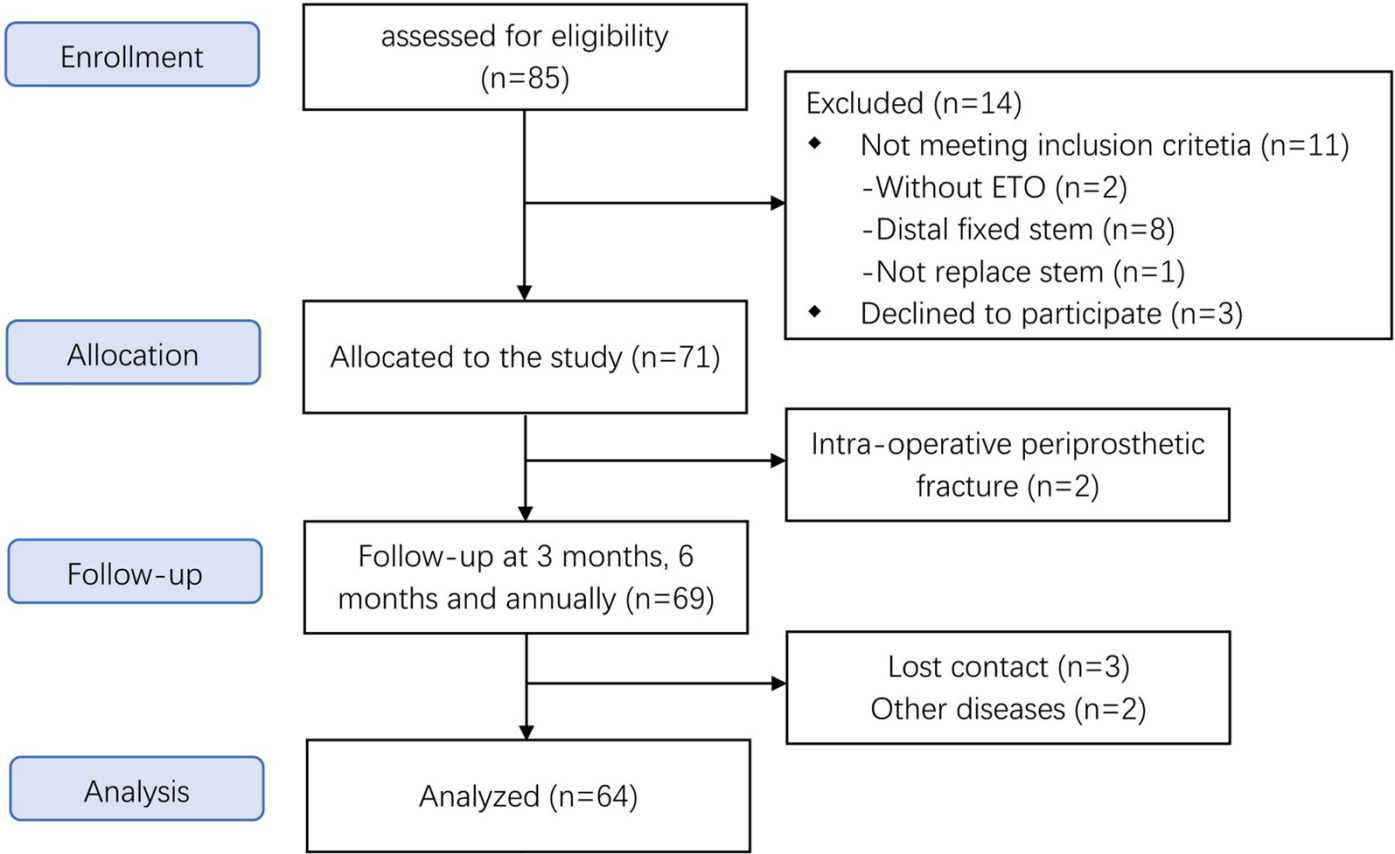
Flowchart indicating number of hips (patients) in the initial and final cohorts. Sixty-four patients were included in the final analysis

From June 2019 to December 2020, patients were screened for eligibility for enrollment in the study using hip radiography and initial screening of medical history. Patient enrollment is depicted in Fig. 1. Patients were eligible for inclusion if they (1) were > 18 years old; (2) required revision hip arthroplasty with ETO; (3) received the Corail revision stem; and (4) signed an informed consent form. The exclusion criteria were (1) lower limb malformation; (2) abnormal bone metabolism; (3) neuromuscular disease of the lower limbs; and (4) inability to cooperate with follow-up. During rTHA, we performed ETO in such patients for the following indications [2]: removal of well-fixed cemented or uncemented stems and cement mantle, correcting varus deformity of the proximal femur, induction of bone healing and ingrowth, trochanteric osteolysis in the presence of metallosis, periprosthetic fractures, improving exposure to the acetabulum, and adjusting the tension in the abductor mechanism.

Sixty-four patients at revision THA were finally followed up. The study group consisted of 29 males and 35 females with a mean age of 60 ± 17 years (range 25–85). The right side was affected in 30 of 64 hips. The mean body mass index was 26.8 kg/m^2^. Demographics and morphological data are presented in Table 1.

**Table 1:** Pre-revision demographics and morphological data

Variables	Initial Cohort (*n* = 64 Hips)
	*n* (%) or Mean ± SD (Range)
Demographics	
Male/Female†	29 (45%)/35 (55%)
Left/Right†	34 (53.13%)/30 (46.87%)
Age at surgery (y)*	60.4 ± 17.58 (25–85)
BMI*	26.8 ± 4.66 (19.48–34.60)
ASA score*	1.8 ± 0.77 (1–3)
1	26
2	24
3	14
Number of previous surgeries†	
1	10 (16%)
2	49 (76%)
3 or more	5 (8%)
Previous THA	
Reason for primary THA†	
Osteoarthritis	21 (33%)
Femoral fracture	12 (19%)
Osteonecrosis	31 (48%)
Primary stem†	
Cemented/Uncemented	14 (21.88%)/50(78.12%)
Revision THA	
Paprosky femoral defect type†	
1	6 (9%)
2	24 (38%)
3A	27 (42%)
3B	7 (11%)
Reason for revision THA†	
Periprosthetic joint infection	55 (86%)
Aseptic loosening	6 (9%)
Recurrent dislocation	3 (5%)
Stem size used in revision†	
11	4 (6%)
12	10 (16%)
13	21 (33%)
14	13 (20%)
15	10 (16%)
16	6 (9%)

*the values are given as the mean and standard deviation

†the values are given as the number with the percentage in parentheses

SD, standard deviation

BMI, body mass index

ASA, American Society of Anesthesiologists

THA, Total hip arthroplasty

### Surgical technique

**Fig. 2:**
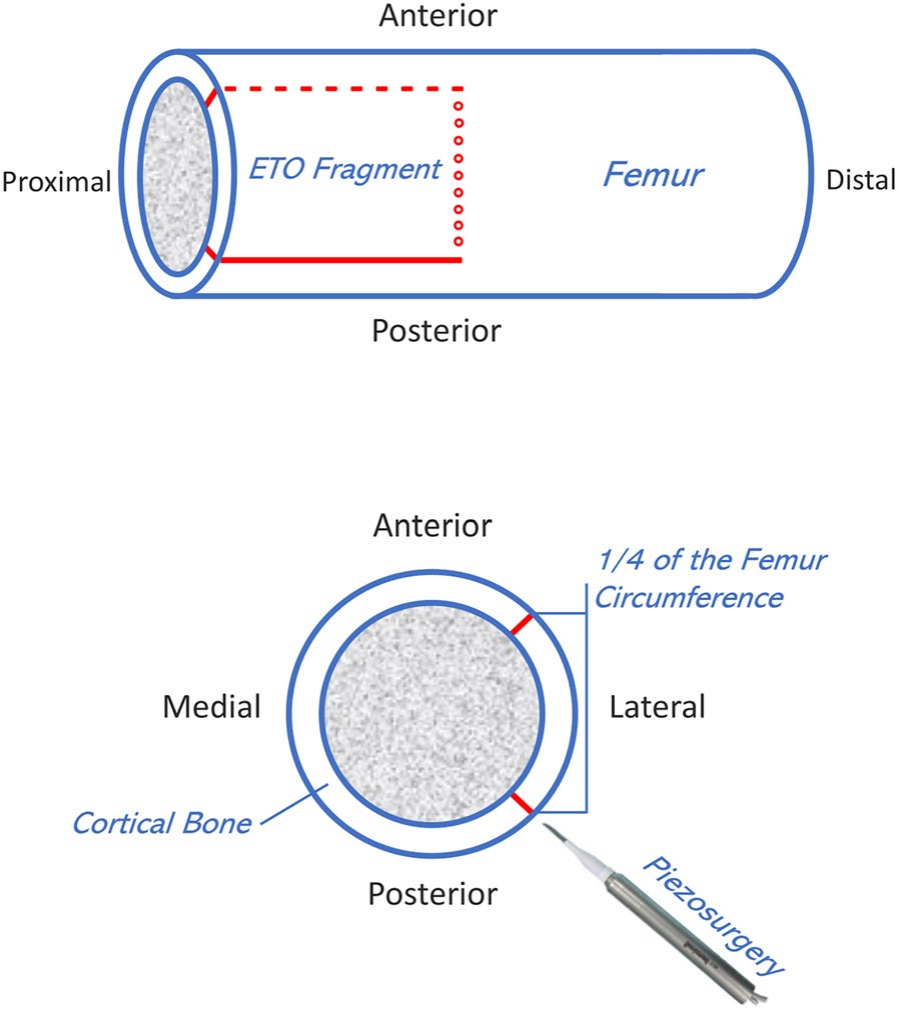
Illustrations depicting an ETO that we have modified

All patients were implanted with Corail revision stem with collar. Preoperative templating was performed by the surgeon to prepare the appropriate stem and ETO planning. The patients with periprosthetic infection treated with two-stage revisions and chronic suppressive antibiotics until the infection was eliminated, and ETO was performed in the first stage. The exact surgical technique of ETO has previously been described in the literature [12, 24]. We modified previous technique slightly to accommodate the implantation of this stem. The extent of ETO was actually determined by the implant to be revised, the presence and size of osteolytic lesions, the position and quality of the femoral isthmus, and the presence of cement. On this basis, the width of the osteotomy fragment should be as narrow as possible, avoiding more than 1/4 of the femur circumference to retain support of the medial femur. For the Corail revision stem, the ETO must not extended below the level where the longitudinal slot of the implanted prosthesis will sit. We tend to perform the posterior osteotomy using a piezosurgery, followed by distal drilling of the lateral cortex. During the osteotomy, the piezosurgery was perpendicular to the femoral cortex to make the fragment wedge-shaped in the cross section, which was conducive to refixation of the fragment. The anterior cortex is osteotomized by using broad osteotomes, and the fragment is finally lifted up to maintain vastus lateralis attachments and vascular supply to the osteotomy fragment because the healing potential of an ETO is dependent on the vascularity of the fragment [2] (Fig. 2). We placed a prophylactic wire distal to the osteotomy site to prevent propagation of fractures distal to the osteotomy. The reamers are used to calibrate the distal cavity of the femur. A small trial stem is implanted to support the fragment. The osteotomy fragment needs to be fixed by cables or wires. It is well documented that this provides sufficient stability of the fragment while minimizing trauma to the soft tissue [7, 25–27]. The broaches are used to access good cortical bone while removing cement and/or debris and reshaping the metaphyseal region to a quadrangular envelope. In this process, the medial cortex of the femur must be protected. The stem was implanted at 10° to 15° anteversion relative to the posterior condylar line (refer to the tibia intraoperatively), irrespective of the preoperative femoral anteversion. Axial and rotational stability was examined using the trial stem. The length of the affected limb was checked. A suitable stem was implanted using the mixed distally and proximally fixed and fit-and-fill principle to ensure initial stability.

All patients were half weight-bearing with crutches in the early postoperative period and were tolerate of progressive weight-bearing by X-ray evaluation at 6–8 weeks postoperatively. The postoperative patient took aspirin orally for 6 weeks to prevent deep vein thrombosis.

### Evaluation

Clinical data for all patients were prospectively collected at the time of revision THA. Patients returned to the hospital for follow-up at 3 months, 6 months after surgery and annually thereafter. Multicenter study records are stored in a secure database. All measurements and data were analyzed by one senior orthopedic surgeon who did not participate in the surgeries. The main observation indexes were osteotomy fragment healing, stem stability and bone remodeling in the femur. Clinical outcomes were assessed with the Harris hip score (HHS) [28], thigh pain, Trendelenburg sign, and patient satisfaction. All patients underwent preoperative and postoperative radiological evaluations including anteroposterior and lateral views of the hip and femur. Radiographic union was assessed by the presence of callus bridging the fragment and/or disappearance of the osteotomy line on orthogonal radiographs [12]. The Engh score was used in predicting the stability of a cementless femoral component [29]. Spot welds refer to new bone formation bridging the stem surface and the endosteal bone [17]. Radiolucent rays were defined as a gap > 1 mm at the prosthesis–bone interface. Considering the possible migration of the greater trochanter through the osteotomy, stem subsidence was evaluated by the difference in the distance from the stem shoulder to the lesser trochanter between the postoperative and the last radiographs [30]. The femur was divided into 14 zones according to Gruen et al [31]. Proximal femur restoration in residual defects areas was subjectively evaluated according to Bohm and Bischel’s criteria [32]. All measurements were performed using OrthoView imaging software (Materialise, Ann Arbor, MI). All measurements were scaled (reference: the diameter of the femoral head in X-ray compared to the actual diameter) to determine the true value.

### Statistical analysis

All statistical analyses were performed by SPSS version 22 (Inc., Chicago, IL, USA). Descriptive statistics are shown as the mean ± standard deviation (SD) (normal distribution), ranges, and proportions. Measurement data were analyzed by Student’s t tests or rank-sum tests. Count data were analyzed by the chi-squared test (where appropriate Fisher’s exact test was alternatively applied). The confidence level for rejecting null hypotheses was set at 95% (*P* < 0.05).

## Results

**Fig. 3:**
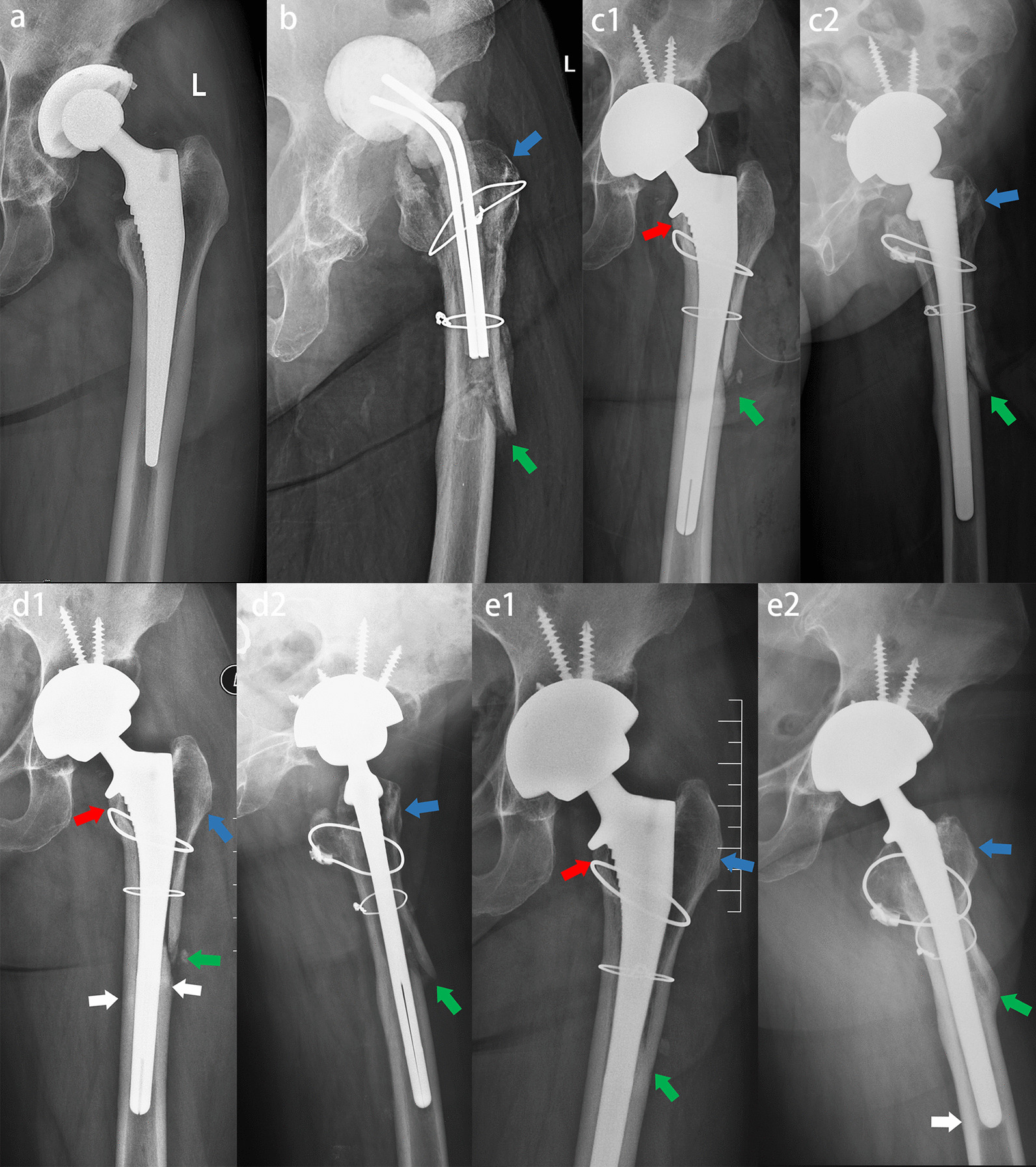
Female, 55 years, periprosthetic joint infection. **a** Preoperative X-ray showed that the stem was well fixed and was removed by using ETO. **b** X-ray two months after spacer implanted showed nonunion of the ETO osteotomy fragment (green arrow) and osteopenia of greater trochanter (blue arrow). **c** The spacer was removed and the Corail Revision femoral stem was implanted. Anteroposterior and lateral X-rays immediately after operation (c1 and c2). The stem and femur matched well. The stem realized immediate and reliable fixation. X-ray showed nonunion of the ETO osteotomy fragment (green arrow) and osteopenia of greater trochanter (blue arrow). **d** Anteroposterior and lateral X-rays two months after operation (d1 and d2) showed spot welds in Gruen3/5 (white arrow), callus in osteotomy line (green arrow) and osteopenia of greater trochanter (blue arrow). **e** Anteroposterior and lateral X-rays three years after operation (e1 and e2) showed no subsidence of the stem, restoration of proximal femur, union of the ETO fragment (green arrow), bone ingrowth from osteotomy fragment to stem (blue arrow), spot welds in femoral isthmus (white arrow), and atrophy of femoral calcar (red arrow)

**Fig. 4:**
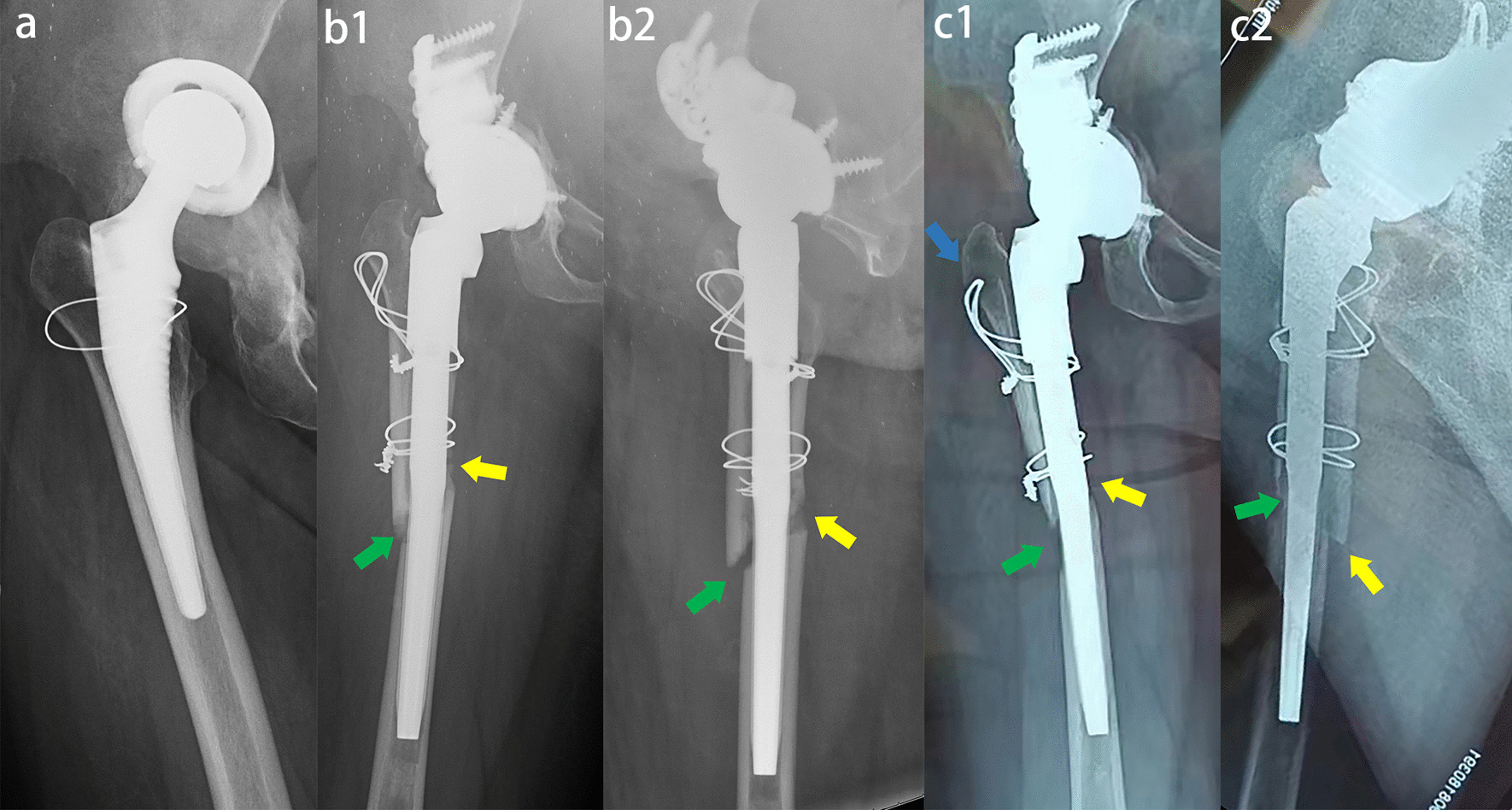
Female, 43 years, aseptic loosening. **a** Preoperative X-ray showed that the stem was well fixed and was removed by using ETO. **b** Anteroposterior and lateral X-rays immediately after operation (b1 and b2). MP stem was used to treat with intraoperative medial femoral cortical fracture (yellow arrow). X-ray showed nonunion of the ETO osteotomy fragment (green arrow). **c** Anteroposterior and lateral X-rays two years after operation (c1 and c2) showed bone atrophy in osteotomy line (green arrow). The medial cortical fragment was nonunion and migrated (yellow arrow). Osteopenia of medial cortical bone due to fracture and loosening

Sixty-nine patients underwent revision hip arthroplasty with Corail revision stem, and radiographs are presented in Fig. 3. Previous to this, two patients had a complication (intraoperative fracture) necessitating use of a modular diaphyseal fitting stem (MP stems, Link, Hamburg, Germany) and were excluded from the study. In both patients, we also obtained postoperative radiographs (Fig. 4).

### Clinical score evaluation

Sixty-four patients were successfully followed up, with an average follow-up time of 34.5 months (23–41 months). Three patients were lost to follow-up, and two could not complete the follow-up because of cancers and stroke. By the end of the follow-up, no serious complications had occurred in any of the patients. The preoperative HHS was 40.7 ± 16.67, and the follow-up HHS was 82.1 ± 6.83. The difference indicated a significant improvement in pain and function. Two (3%) patients had transient thigh pain in the early postoperative period, but thigh pain did not recur at the last follow-up. Fifty-two (81%) patients returned to normal gait. Three (5%) patients presented a positive Trendelenburg sign. Almost all (98%) patients were satisfied with the clinical outcome (Table 2).

**Table 2:** Post-revision clinical outcome of the final cohort

Variables	Cohort at Final Follow-Up (n = 64 Hips)
	n (%) or Mean ± SD (Range)
Follow-up (m)*	34.5 ± 4.95(23–41)
HHS*	
Pre-operation	40.7 ± 16.67 (0–67)
Post-operation	82.1 ± 6.83 (73–93)
Thigh pain †	2 (3%)
Gait†	
Limp	12 (19%)
Normal	52 (81%)
Trendelenburg sign†	
Positive	3 (5%)
Negative	61 (95%)
Satisfaction†	
Very satisfied or satisfied	56 (87%)
Somewhat satisfied	7 (11%)
Somewhat dissatisfied or dissatisfied	1 (2%)

*the values are given as the mean and standard deviation

†the values are given as the number with the percentage in parentheses

*SD* standard deviation

*HHS* Harris Hip Score

### Radiographic evaluation

**Fig. 5:**
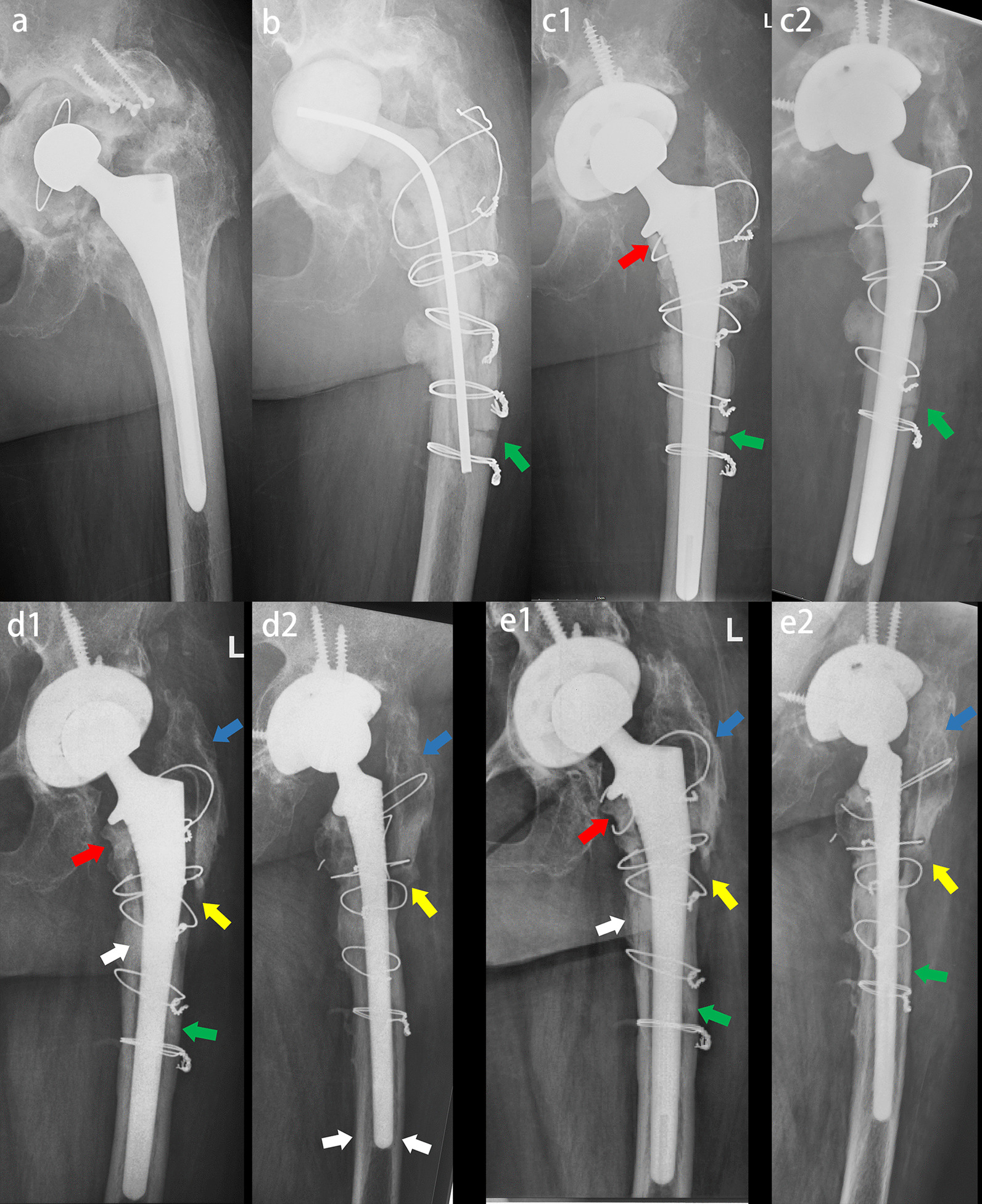
Female, 69 years, periprosthetic joint infection. **a** Preoperative X-ray showed that the stem was well fixed and was removed by using ETO. **b** X-ray four months after spacer implanted showed nonunion of the ETO osteotomy fragment (green arrow). The wires were embedded in the femoral cortex. **c** The spacer was removed and the Corail Revision femoral stem was implanted. Some of the wires were replaced. Anteroposterior and lateral X-rays immediately after operation (c1 and c2). The stem and femur matched well. The stem realized immediate and reliable fixation. X-ray showed nonunion of the ETO osteotomy fragment (green arrow). **d** Anteroposterior and lateral X-rays one year after operation (d1 and d2) showed spot welds in Gruen3/5/6 (white arrow), callus in osteotomy line (green arrow) and osteopenia of greater trochanter (blue arrow). The osteotomy fragment was fractured and migrated (yellow arrow). **e** Anteroposterior and lateral X-rays three years after operation (e1 and e2) showed the stem stability, union of the ETO fragment (green arrow), spot welds in femur (white arrow), atrophy of femoral calcar (red arrow). Osteopenia of greater trochanter due to fracture and loosening (blue arrow)

Sixty-two patients who underwent ETOs achieved complete healing at the final follow-up, 59 of whom had bony ingrowth from the osteotomy fragment to the stem without radiolucent lines. The Engh score was 21.3 ± 3.59 (15.5–27.0) on X-ray. There were 7 hips (11%) with radiolucent lines, which were found in the proximal Gruen1, Gruen 2 and Gruen7 regions without progression. Spot welds were observed in 29 patients, predominantly in the femoral isthmus distal to the ETO (Table 3). Evidence of proximal femoral bone remodeling was observed as early as one year postoperatively. In the recent follow-up, 43 patients showed evidence of proximal femoral bone mass recovery. We observed evidence of proximal stress shielding in five patients, without progression after one year. Two patients had fractures of the osteotomy fragment because of osteoporosis that failed to heal, without revision (Fig. 5).

**Table 3:** Post-revision radiographic outcome of the final cohort

Variables	Cohort at final follow-Up (n = 64 Hips)
	n (%) or Mean ± SD (Range)
ETO†	
Union	62 (97%)
Fracture	2 (3%)
Engh score*	21.3 ± 3.59 (15.5–27.0)
Fixation	6.7 ± 2.67 (2.5–10.0)
Stability	14.6 ± 1.79 (11.5–17.0)
Hips with radiolucent lines†	7 (11%)
Gruen zone 1 only	4 (6%)
Gruen zone 7 only	2 (3%)
Gruen zones 1 and 2	1 (2%)
Spot welds†	29 (45%)
Gruen zone 3	15 (23%)
Gruen zone 5	7 (11%)
Gruen zone 6	2 (3%)
Gruen zone 3 and 5	5 (8%)
Proximal femur Remodeling†	
Regeneration	43 (67%)
No Change	16 (25%)
Stress Shielding	5 (8%)

*the values are given as the mean and standard deviation

†the values are given as the number with the percentage in parentheses

*SD* standard deviation

*ETO* extended trochanteric osteotomy

The mean subsidence of the stem was 1.8 mm (range 0–4 mm) (Table 4). A significant finding was that the stem subsidence was significantly less in collar-contact-calcar patients than in non-collar-contact-calcar patients (*P* = 0.03). The stem subsided within three months postoperatively and stabilized one year later. No patient developed symptoms of stem loosening.

**Table 4:** Subsidence of stem

Variables	Subsidence distance
	Mean ± SD (Range)
Subsidence in all stem (mm)	1.8 ± 1.41 (0–4)
Subsidence in collar-contact-calcar stem	1.4 ± 1.25 (0–3)
Subsidence in not-collar-contact-calcar stem	3.0 ± 1.20 (1–4)
P	0.03

*SD* standard deviation

## Discussion

In this study, our findings show that the Corail revision stem provides functional benefit with 100% survival to rTHA patients undergoing ETO. The postoperative HHS at the last follow-up was significantly higher than the preoperative HHS. The healing rate of ETO was 97% in the patients who used Corail revision stems. Two years after surgery, radiologic signs of bony support to the stem were observed in most patients. Restoration of the proximal femur occurs in a “up to down” direction. The stability of the stem was ensured by excellent bone ingrowth and remodeling. The collar effectively reduces the subsidence of the stem. Most patients returned to normal gait, and the satisfaction rate was very high. However, these ideal results are dependent on an intact medial femoral cortex.

Femoral components that achieve diaphyseal fixation are currently a popular therapeutic option in prosthetic femoral revision surgery, especially in the presence of extensive proximal bone loss [33]. The longer revision stems are designed to bypass bone loss proximally by achieving press-fit distally and offer the surgeon remarkable advantages regarding implant stability, axial and rotational implant control, and leg length control with high survivorship [6, 7]. Some scholars have reported that the use of such prostheses is associated with severe postoperative thigh pain (8–9%) [6, 34] and severe stress shielding and bone loss the proximal femur (22–50%) [15–17]. These result in an inability to achieve the main objectives of femoral revision, which include achieving long-term implantation and fixation, improving patients’ quality of life, reducing the incidence of complications, maintaining or restoring the bone mass of the proximal femur, providing a foundation for future surgery, and creating a biomechanically restored hip [34].

Corail revision stem had an excellent clinical outcome and avoided some complications with distally fixed stem. Corail revision stems are generally longer than primary stems, maximize the surface area for bone growth to achieve fixation in patients with poor bone stock and require use of mixed distally and proximally fixed and fit-and-fill principle, thereby reducing the risk of aseptic loosening [23]. This stem achieves axial and rotational stability, which is a prerequisite for successful femoral revision [35].The 100% survival rate and improved HHS score in this study confirmed that the Corail revision stem can achieve the main objectives of femoral revision. Corail revision stems avoided thigh pain occurring in distally fixed stems. At the same time, the cost of Corail Revision stem is only 3600 USD, which is much lower than that of modular revision stem, such as MP stem, which is 5600 USD.

In our study, all patients undergoing revision required ETO for removal of the stem and requiring early healing at the osteotomy. ETO is considered the gold standard technique for the removal of a well-fixed stem, and can be applied to nearly any patient undergoing femoral revision [27]. Previously, Saunders et al [22] reported in their study that 30 hips with ETO healed well. Therefore, they believe the Corail revision stem is safe when the distal end of the stem is at least 4 cm from the ETO. We operated on the ETO osteotomy level above the longitudinal groove of the stem. Sixty-two patients experienced ETO union. Compared with stress shielding that is common with distally fixed stems, bone ingrowth occurred between the osteotomy fragment and the Corail revision stem, which avoided stress shielding and promoted the healing of the osteotomy. Two hips had nonunion of the ETO. We thought the reason was the fixation of the ETO with wires in elderly patients with osteoporosis, which was also confirmed in previous studies [36]. In the future, we will use claw plate for such patients with osteoporosis [37].

It is worth noting that postoperative radiographs showed bone ingrowth occurring at the proximal femur. The ability of this stem to promote bone growth and re-establish bone stock could be attributed to the HA coating on the entire stem surface without any bone grafting. Many studies on HA coatings have specifically addressed femoral component revision, and HA coatings should potentially increase bony ingrowth and minimize stress shielding [22, 23, 35, 38, 39]. Early and extensive bone deposition over HA coatings has been verified in postmortem analyses compared with porous and grit-blasted coatings [40]. Stress stimulation plays an important regulatory role in cell proliferation at the fracture end, and the formation of blood vessels and callus, provides rich blood supply to the fracture end, and promotes the growth of callus and the formation of new bone [41]. In contrast, stress shielding in distally fixed stems leads to severe bone loss in the femoral metaphysis and proximal diaphysis, which can also challenge adequate fixation of the femoral stem.

In this study, we found that the collar played an important role in the stability of the prosthesis. In collar-contact-calcar hips, the subsidence of the stem is significantly less than that in non-collar-contact-calcar hips. Collared stems have been suggested to promote immediate stem stability, resulting in lower subsidence and revision rates than collarless stems [42]. Demey et al. [43] found that collared uncemented stems have significantly greater immediate stability than collarless stems. Johnson et al. [44], in a paired cadaveric biomechanical analysis, showed that collared stems seemed to offer a protective effect in torsional loading in this biomechanical model. Thus, the collar stem is not inferior to the distally fixed tapered stem in the initial stability of the prosthesis.

Corail revision stem cannot be used in patients with medial cortical bone destruction, which plays a crucial role in the stability of the prosthesis. Once the medial cortical bone is damaged, this stem will not be able to achieve three-point fixation. In this case, only the distal fixed stem can be selected. Based on our experience and practice with this stem, we raise the following suggestions. First, the medial cortical bone should be carefully protected during the operation. Second, multiple cables or wires should be fixed to the fragment for stability. In patients with severe osteoporosis, a claw plate is required. Third, on the basis of removing original stem, the length of ETO should not be too long to avoid inadequate distal fixation.

There are also some limitations in our study. First, the number of patients implanted with the Corail revision stem was small because this stem was not widely used in rTHA with ETO. Second, our evaluation was performed only radiographically, not histologically. However, histological examination is actually difficult. So, our evaluation is appropriate and this is a weak limitation. Third, all patients underwent a short-term follow-up, with an average time of 34 months. The follow-up time seemed to be adequate, as bone restoration can mainly be expected to occur within two years. Despite these limitations, the study suggests that Corail revision stem can be safely used in rTHA with ETOs without compromising the ETO union, while preserving the proximal femur mass.

## Conclusion

Our study shows that the Corail revision stem is a viable and reliable option in rTHA patients with ETO. This stem was reported to have excellent clinical and radiographic outcomes, resulting in a high rate of ETO union and stem survival. The revision stem enabled restoration of proximal bone stock in femurs with prerevision bone defects, which were prepared for the next revision operation. However, this stem can only be used in rTHA patients with an intact medial femoral cortex.

## Data Availability

The datasets used and/or analyzed during the current study are available from the corresponding author upon reasonable request.

## Abbreviations

rTHA: Revision total hip arthroplasty
ETO: Extended trochanteric osteotomy
HHS: Harris hip score
HA: Hydroxyapatite
